# Proposed identification of physiological classification and theoretical mechanisms of *yogāsanas*

**DOI:** 10.1016/j.jaim.2021.06.024

**Published:** 2021-12-20

**Authors:** Samiran Mondal

**Affiliations:** Department of Yogic Art & Science, Visva-Bharati (Central University), Santiniketan, 731235, West Bengal, India

**Keywords:** Classification of *Yogāsana*, Mechanism of *Yogāsana*

## Abstract

Yoga in the theoretical and practical form is now accepted all over the world, by the researchers as well as by the general population. *Yogāsana* or Yogic postures are one of the main foundations of practical yoga. Mechanisms of Mediation and *Prānāyāma* has already been established. In this article, the author is for the first time proposing physiological classification of *Yogāsana* i.e. (a) Stretch (b) Contraction (c). Neuromuscular Coordination (d) Sense Reduction (e) Spine Brain Activation. In support of this physiological classification author has proposed five hypothetical theories i.e. (i) Stretch Relaxation Awareness (ii) Contraction Relaxation Awareness (iii) Ballastic Contraction Relaxation Awareness (iv) Sensory Motor Function Awareness and (v) Vital Energy Flow Awareness to understand the underlying mechanisms of *Yogāsana* practice and to explain its benefits.

## 1 Introduction

### 1.1 Yoga and Yogasana (Yogic posture)

Yoga is an ancient *Bharatian* (Indian) and *Hindu* culture. Archaeological evidence of Yoga seals (3300 BC) were excavated from the Harrapan civilization or Indus valley civilization [1], [2], [3], [4], [5], [6]. The *Vedas*, world's oldest literature (2500 BCE) and *Upanishads* eluminated the wisdom of *Yoga* from the time of Vedic period [7,8]. *Yoga* culture was an essential part of *Vedic* *tapabana* *ashramic* educational system and in daily life from that time. *Ramāyana* and *Mahābhārata* the historical marvel of India recognised the practice and benefits of Yoga in *Chaturāśhramas* of life: *Brahmacharya*-(celibate student hood and life building time, up to the age 25 years); *Gārhasthya* (house holder stage and family life up to the age of 50 years); *Vāṇaprastha* (detachment from the family life, up to the age of 75 years); and (complete detachment and surrender to the God, after the age of 75 years). In the time of *Mahābhārata, Srīmadbhagabadgītā*, the conceptual gist of *Vedas* and *Upani*ṣ*hads,* was written with all its 18 chapters' names attached with *Yoga*. It was the first time, *Yoga* was elaborated for general population and through it the general population received direction to cope with this world. *Gītā* described the four main paths of life: *J*ň*āna Yoga* (Wisdom seeker); *Bhakti Yoga* (Devotion maker); *Karma Yoga* (Selfless worker) and *Dhyāna Yoga* (Practical experiencer) to achieve success and peace in life. *Takshashila* university (600 BCE), the world's oldest University and *Nalanda*, another ancient university of India introduced compulsory *Yoga* in all their educational curriculums. During and after *Bauddha* and *Jain* period (500 BCE) *Yoga* was accepted by all spheres of life and people practiced *Yoga* regularly for their physical, mental, and spiritual wellbeing.

Ayurveda, the world's oldest record of medical practice [2,6] is ancient Indian medicinal system. Ayurveda meaning the science of long and healthy life, describes human being as the combination of body, mind, and soul. It prescribes *Vyāyama* (exercise) for the body and *Yoga* for mind and soul [9,10]. However, in the Ayurvedic text *Yoga* has been discussed only with few *slokes* (opharisms) and strongly recommended it as a specialized area [11]. *Dhyāna Yoga* or *Rāja Yoga* was explained in a structured form in *‘Yoga Sutra’* written by the Sage Patanjali [12]. In this text had been described in eight limbs (*Astāṅga Yoga*): *Yama* (Social discipline); *Niyama* (Individual discipline); *Āsana* (Yogic postures); *Prāṇāyama* (Control of the vital energy through breathing); *Pratyāhāra* (Withdrawal of sense organ); *Dhāraṇā* (Concentration); *Dhyāna* (Deep concentration) and *Samādhi* (Deepest concentration). The detail description of *Yogasāna* *(Yogic postures)*, the third limb of Astranga Yoga was found in the ancient Hatha Yogic text: *Hatha Yoga Pradīpikā* [13]; *GhrendaSamhitā* [14]; *Śhiva Samhitā* [15]; *Vaśiṣṭhasaṁhitā* [16] and *Haṭharatnāvalī* [13] with the description of eighty four *yogāsanas*. Scientific description with classification on *Yogāsana* has been available in the modern literature [[17], [18], [19]]. In the most authentic way, Iyengar [20] described with two hundred *Yogic* postures. Both the ancient and modern literature on *Yogāsana* claim that it improves wellbeing and can be effective for therapeutic purposes. The aim of this article is to propose physiological classification and theoretical mechanisms to understand *Yogāsana*, in its practices.

### 2 Proposal of classification of Yogasana: On the basis of physiological functions

*Yogāsana* have been classified by the authorities as meditative and cultural postures including relaxation posture [17]; along with meditation, standing, back bending, forward bending, spinal twisting, inverted, balancing poses [18]; standing, sitting, prone, supine, topsyturvy and relaxation postures [19]; and primary, intermediate and advance postures [20]. The author is actively involved in Yoga research for past 30 years and guided three PhD projects [[21], [22], [23]]. Also the author has published many research papers on scientific Yogāsana practice protocol [[24], [25], [26], [27], [28], [29]]. With these experiences, author world like to propose new *Yogāsana* (Yogic postures) classification on the basis of physiological functions, i.e: (a) Stretch (*Paschimottanāsana* etc.); (b) Contraction (*Mayūrāsana* etc.); (c) Neuromuscular Coordination (*Vṛkṣāsana* etc.); (d) Sense Reduction (*Śavāsana* etc.); (e) Spine Brain Activation (*Padmāsana* etc.). However, in the practice of *Yogāsana* three principles (i.) Slow movement awareness, (ii.) Body awareness and (iii.) Breathing awareness should be present in a synchronize manner to achieve the best result [[17], [18], [19], [20]].

### 3 Proposal of five theories: theoretical mechanisms of *Yogasana*

Earlier Srininvasan [30] and Murphy [31] tried to understand *Yogasana* (Yogic postures) in the perspective of Proprioceptive Neuromuscular Facilitation (PNF) technique. PNF is very useful scientific technique for improving the daily life activities, injury/illness prevention and rehabilitation. The PNF scientists had proposed four theoretical mechanisms: i. Autogenic Inhibition; ii. Reciprocal inhibition; iii. Stress relaxation; iv. Gate control theories [[32], [33], [34], [35]]. Here, the author would like to propose five theories to understand the underlying mechanisms of Yogāsana(Yogic posture): i.e. i. Stretch Relaxation Awareness; ii. Contraction Relaxation Awareness; iii. Ballastic Contraction Relaxation Awareness; iv. Sensory Motor Function Awareness; and v. Vital Energy Flow Awareness for discussion and suggestions from the scientific community.

#### 3.1 Stretch relaxation awareness theory

The author proposes that some asanas can be classified as ‘Stretch’ for example *Paścimottāsana*, *Bhujaṅgāsana*, *Śalabhāsana* etc. At the time of stretching muscles spindle (stretch receptors) could be activated and after releasing these asanas same area would be relaxed than before. These functions will activate sensory and motor cortex. Body and breathing awareness will activate more brain parts to be engaged in this process. So, the concentration is developed automatically to maintain these postures.

#### 3.2 Contraction relaxation awareness theory

In this theory, golgi tendon organ (GTO), another mechanoreceptor is targeted. Some Yogāsanas can be grouped as ‘Contraction’ for example *Mayūrāsana*, *Utkatāsana* etc. At the time of performing these asanas fullest contraction could be focused on the related muscles. As a result, the GTO is activated and after releasing same area is relaxed much more. This process also activates the sensory-motor cortex. At the same time body and breathing awareness activates many other regions of the brain and enables concentration.

#### 3.3 Ballistic contraction relaxation awareness theory

In this theory, the author would like to highlight vestibular apparatus in the inner ear and pacinian corpuscles present in the skin. Some balance or ‘Neuromuscular Coordination’ *Yogāsana* like *Vṛkṣāsana*, *Garuḍāsana*, *Natarājāsana* etc. could activate these two receptors with their related sensory motor and other cortical areas. Also these type of asanas should activate lower brain or Cerebellum for coordination and body awareness. The Pons and Medulla areas are also involved for monitoring, the breathing awareness. Together, these activations may develop more concentration because many parts of the brain are involved in these balance type of *Yogāsana*.

#### 3.4 Sensory motor function awareness theory

In the fourth theory, the author proposes relaxation āsanas or ‘Sense Reduction type’ asanas such as *Shavāsana, Advāsana, Makarāsana* etc. Here, the sensory motor nerve activity is forcefully reduced. So, the sensory-motor awareness in the cortical areas is slowly diminished. Also, by detracting and diverting all the external and internal sensory motor organs' activity these types of asanas could produce relaxation feelings.With this breathing awareness makes the person more aware about the vital activity of the body and beyond. Specially, the Midbrain area i.e. the filtering unit of sensory motor information has been deactivated and feeling of relaxation is persisted.

#### 3.5 Vital energy flow awareness theory

The fifth proposed group in this article is ‘Spine-Brain Activation’. In this group of asanas also called as meditative posture like *Padmāsana*, *Śukhāsana*, *Siddhasana*, and *Svastikāsana* etc. Here, the spine and head should be erect to allow all neural impulses and vital energy to flow smoothly from the lower part of the spine to all areas of the brain and back to the lower part again. In these type of meditative postures, all the somatic and subtle autonomic neural awareness and vital organ awareness are subsided. Brain and spine level electrical activity and energy cost is reduced. But the higher cortical activity increased. Also in these type of asanas one may feel the *PranaVayu* (vital energy) in the different parts of the body and *Chakras* (energy wheels) in the spine and brain areas. One can relate individual energy with cosmic energy.

## 4 Conclusion

*Yoga* with all its forms (practical and theory) is now accepted globally. *Yogāsanas* or *Yogic* postures are one of the pillars of the practical *Yoga* foundation. Analysis of mechanisms of meditation and *prāṇayāma* has already been established. *Yogāsana* effect in the human psychophysiological areas and its underlying mechanisms are not yet been identified. In this article, the author classified *Yogāsana* from the physiological point of view i.e.Stretch, Contraction, Neuromuscular Coordination, Sense Reduction and Spine Brain Activation for the first time. To support the physiological classification the author has also for the first time proposed five theories i. Stretch Relaxation Awareness ii. Contrction Relaxatation Awareness iii. Ballistic Contraction Relaxation Awareness iv. Sensory-Motor function awareness and v. Vital Energy Flow Awareness to understand the underlying mechanisms of *Yogāsna* practices. All these hypothetical theories should be examined by scientific protocols and methods for final conclusion.

## Conflict of Interest

The author declare that there is no conflict of interests regarding the publication of this paper.
